# Effects of probiotic *Lactobacillus brevis* KB290 on incidence of influenza infection among schoolchildren: an open-label pilot study

**DOI:** 10.1111/lam.12340

**Published:** 2014-10-30

**Authors:** N Waki, M Matsumoto, Y Fukui, H Suganuma

**Affiliations:** Research and Development Division, Kagome Co., Ltd.Nasushiobara, Tochigi, Japan

**Keywords:** incidence, influenza, *Lactobacillus brevis* KB290, probiotics, schoolchildren

## Abstract

**Abstract:**

We investigated the efficacy of dietary consumption of *Lactobacillus brevis* KB290 (KB290) against influenza in humans by a preliminary intervention study on elementary schoolchildren, using a commercially available probiotic drink. Subjects were divided into Groups A and B, and an open-label, parallel-group trial was conducted in two 8-week periods at a 1-month interval in winter 2013/2014. Group A was provided with a bottle of the test drink containing KB290 (about 6 billion colony-forming units) every school day in the first period and had no treatment in the second period, and *vice versa* for Group B. Epidemic influenza was not observed during the first period and only two of 1783 subjects were diagnosed. In the second period, the incidence of influenza in Groups A (no treatment) and B (provided the test drink) was 23·9 and 15·7%, respectively, and the difference was statistically significant (*P *<* *0·001). The reduction in the incidence of influenza by KB290 consumption was especially remarkable in unvaccinated individuals. This is believed to be the first study to show a probiotic food reducing the incidence of influenza in schoolchildren, although further studies are needed to confirm the effectiveness of the probiotic strain KB290.

**Significance and Impact of the Study:**

We demonstrated a reduction in the incidence of influenza in 1089 schoolchildren by continual intake of a probiotic drink containing *Lactobacillus brevis* KB290 (KB290), isolated from a traditional Japanese pickle ‘Suguki’. The effect was especially evident in subjects not inoculated with influenza vaccine. This is believed to be the first report to show reduced incidence of influenza in schoolchildren taking a probiotic food. Further studies are needed to confirm the effectiveness of the probiotic strain KB290, which may be useful in the development of potential anti-influenza agents derived from common foods.

## Introduction

Influenza is a respiratory infection caused by influenza virus (IFV), which produces a variety of symptoms including high fever, chills, sore throat, headache, runny or blocked nose, weakness, muscle pain and sometimes diarrhoea (Barik 2012). These clinical symptoms often become severe in elderly individuals and infants because of a poor or weakened immune system (Guillemard *et al.* 2010; Gasparini *et al.* 2013). Vaccination against IFV is usually administered as a practical, prophylactic method, but is not necessarily sufficient because viral mutagenesis occurs rapidly (Ujike *et al.* 2010). Therefore, it is desirable to boost the immune system and promote resistance against IFV infection in daily life.

*Lactobacillus brevis* KB290 (KB290) is a plant-derived lactic acid bacterium (LAB) isolated from ‘Suguki’, a traditional pickle produced in Kyoto, Japan. KB290 has been reported to be safe for human consumption; it is tolerant to gastrointestinal juices, improves gut health (Nobuta *et al.* 2009) and is useful for early intervention in irritable bowel syndrome (Murakami *et al.* 2012). In addition, it has been revealed that orally administered KB290 enhances interferon (IFN)-*α* production in humans (Kishi *et al.* 1996) and increases cell-mediated cytotoxic activity of splenocytes in mice (Fukui *et al.* 2013, 2014). We have previously reported that oral administration of KB290 could alleviate IFV-induced clinical symptoms in mice (Waki *et al.* 2014) and that these effects of KB290 may have been elicited by long-lasting enhancement of IFN-*α* production and augmentation of IFV-specific immunoglobulin A production. These findings indicate that oral administration of KB290 may help reduce the risk of influenza in humans.

Some LAB strains have been reported to reduce the incidence of respiratory symptoms such as fever, rhinorrhoea and coughing (Leyer *et al.* 2009), and to decrease the duration of common infectious diseases including respiratory tract and gastrointestinal tract infections (Guillemard *et al.* 2010). However, a limited number of studies have evaluated the effects of probiotics on rate of subjects diagnosed with influenza by physicians. In addition, a previous systematic review showed that probiotics may have a beneficial effect on the severity and duration of symptoms but not the incidence of respiratory tract infections (Vouloumanou *et al.* 2009). Thus, we conducted a preliminary, open-label, parallel-group trial to evaluate whether a probiotic drink containing KB290 could reduce the incidence of influenza among elementary schoolchildren.

## Results and discussion

### Recruitment and participant flow

**Figure 1:**
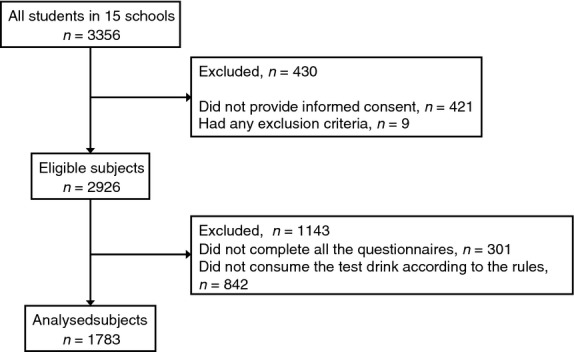
Flow of participants through the study.

The participant flow diagram for this study is shown in Fig.1. Subjects were recruited in the 15 participating schools, which had 3356 schoolchildren in total. We excluded 421 children who did not provide written informed consent from their guardians and nine children who had any of the exclusion criteria listed in the Subjects and Methods section. After exclusion, 2926 subjects were eligible. After follow-up of the trial, we selected 1783 subjects for the statistical analyses, who completed all questionnaires, did not miss consumption of the test drink in the consumption period and never consumed the drink in the nonconsumption period. For this selection, we excluded 301 subjects who did not complete all questionnaires and 842 who did not consume the test drink according to the rules.

### Incidence of influenza

**Figure 2:**
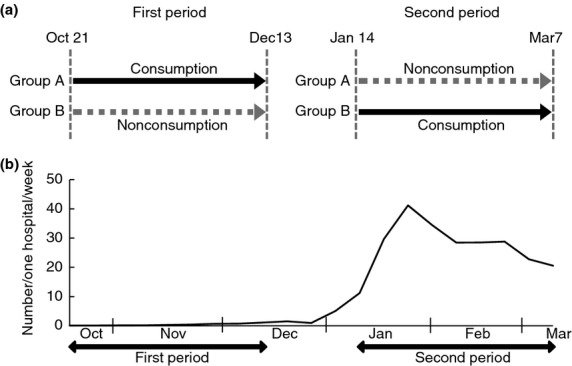
(a) Clinical trial schedule. For subjects in Group A, test drink containing KB290 was provided 5 days per week for 2 months in the first period (consumption period; solid arrow) and no treatment in the second period (nonconsumption period; dotted arrow), while those in Group B were defined *vice versa*. (b) Number of people diagnosed with influenza in Tochigi prefecture. Data were derived from the Tochigi Prefectural Infectious Disease Surveillance Centre (2014).

As described in Fig.2a, the study consisted of two periods: October 21 to December 13, 2013, and January 14 to March 7, 2014. The 15 participating schools were divided into Groups A and B. Subjects in Group A were provided with a bottle of the test drink containing KB290 on every school day (5 days per week) in the first period and not in the second period, while those in Group B were given the drink in the second period and not the first (Fig.2a). The incidence of influenza was compared between the nonconsumption and the consumption groups in each period (Table1). The numbers of analysed subjects of Group A and B were 694 and 1089, respectively. In the first period, no subject was diagnosed with influenza in the consumption group and only two in the nonconsumption group, and there was no significant difference between the two groups. The epidemic of influenza in Tochigi prefecture, where the trial was conducted, was reported to be from the end of January to the beginning of February 2014 by the Tochigi Prefectural Infectious Disease Surveillance Centre (Fig.2b). Therefore, we concluded that the first period was not suitable to evaluate the incidence of influenza. In the second period, when the epidemic occurred, 23·9 and 15·7% of subjects were diagnosed with influenza in the nonconsumption and consumption groups, respectively (Table1), and there was a significant difference between the groups (*P *<* *0·001). This suggests that continual consumption of KB290 during the influenza season could help reduce the risk of infection.

**Table 1:** Incidence of influenza

	Subjects diagnosed with Influenza (*n*/total *n* (%))		RR	95% CI	*P* value
	Nonconsumption group	Consumption group			
First period					
All subjects analysed	2/1089 (0·2)	0/694 (0·0)	–	–	0·259
Second period					
All subjects analysed	166/694 (23·9)	171/1089 (15·7)	0·656	0·542, 0·795	< 0·001
Vaccinated subjects	70/356 (19·7)	70/466 (15·0)	0·764	0·565, 1·032	0·079
Unvaccinated subjects	96/335 (28·7)	101/620 (16·3)	0·568	0·445, 0·727	< 0·001

RR, relative risk; CI, confidence interval.

The incidence among all 3356 schoolchildren belonging to the schools of Groups A and B was 19·8% (325 of 1645 children) and 17·9% (306 of 1711 children), respectively, and no significant difference was observed [relative risk (RR) = 1·105, 95% confidence interval (CI): 0·960–1·272, *P *=* *0·165]. This indicated that the scale of the influenza epidemic in the two groups did not differ significantly. Therefore, the reduction in influenza infection observed in the consumption group could have been due to KB290 consumption, rather than differences in influenza incidence among the schools.

It has been demonstrated that influenza vaccination affects the incidence of influenza in schoolchildren (Sugaya *et al.* 1994; Neuzil *et al.* 2001). Therefore, we stratified the subjects into vaccinated and unvaccinated groups and performed a differential analysis. The reduction in the incidence of influenza by consuming KB290 was not observed in the subjects inoculated with influenza vaccine. In contrast, in the unvaccinated subjects, the reduction in incidence in the consumption group was more significant than that observed in the analysis for all subjects (Table1).

The additive effects of ingestion of probiotics on influenza vaccine immunogenicity have been demonstrated previously (Olivares *et al.* 2007; Davidson *et al.* 2011). In the present study, however, consumption of the test drink containing KB290 was not found to enhance the effect of vaccination. This difference could have been caused by the sufficient effect of the vaccination in the present study. Many previous studies have revealed that vaccination is effective for the prevention of influenza in children (Neuzil *et al.* 2001; Kawai *et al.* 2011). Indeed, in the nonconsumption group in the present study, the incidence of influenza in the vaccinated subjects was significantly lower than that in the unvaccinated subjects (RR = 0·686, 95% CI: 0·524–0·898, *P *<* *0·01), indicating that vaccination was sufficiently effective to prevent influenza in this trial.

### Incidence of common cold or gastroenteritis

We also evaluated the incidence of the common cold or gastroenteritis (Table2). In the first period, the incidence of the common cold in the consumption group was significantly higher than that in the nonconsumption group. In the second period, there was no significant difference in the incidence between the two groups. The incidence of gastroenteritis was low, and there was no difference between the two groups in both periods. KB290 has been reported to enhance cell-mediated cytotoxic activity in mice (Fukui *et al.* 2013, 2014). In addition, Makino *et al*. (2010) have reported that consumption of yoghurt fermented with LAB augments cytotoxic activity and reduces the risk of the common cold. One of the reasons that we could not confirm any significant effect against the common cold was that the diagnostic criteria may have differed with each physician. KB290 might have had an effect on the common cold if we had defined the criteria for it. It could also be that the epidemic scale of the common cold during the trial differed among the schools, unlike the incidence of influenza, because we did not evaluate the incidence of the common cold in all schoolchildren, including nonstudy subjects.

**Table 2:** Incidence of common cold and gastroenteritis

	Subjects diagnosed with common cold or gastroenteritis (*n*/total *n* (%))		RR	95% CI	*P* value
	Nonconsumption group	Consumption group			
First period					
Common cold	131/1089 (12·0)	108/694 (15·6)	1·294	1·021, 1·638	0·033
Gastroenteritis	19/1089 (1·7)	17/694 (2·4)	1·404	0·735, 2·682	0·302
Second period					
Common cold	62/694 (8·9)	117/1089 (10·7)	1·203	0·897, 1·612	0·215
Gastroenteritis	27/694 (3·9)	63/1089 (5·8)	1·487	0·957, 2·310	0·075

RR, relative risk; CI, confidence interval.

### Adverse events

The principal doctor in this trial concluded that there were no adverse events associated with consuming the test drink. Two subjects reported poor health condition such as abdominal pain and deteriorating cold symptoms, but all of these events were transitory, not serious, and did not seem to be related to consumption of the test drink.

The present study suggests that continual consumption of probiotic KB290 in the influenza season may help reduce the risk of infection. To the best of our knowledge, this is the first study to evaluate the effects of probiotics on the incidence of influenza in an interventional trial on a large number of children, although it was an open-label pilot study. Namba *et al*. (2010) reported that ingestion of *Bifidobacterium longum* BB536 reduced the incidence of influenza in elderly, but the number of subjects in the trial was small (*n *=* *27). Another study by Merenstein *et al*. (2010) suggested that consumption of a probiotic drink containing *Lactobacillus casei* DN-114 001 decreased the incidence of common infectious diseases in 638 children, although the effect on influenza was not evaluated. Regarding foods or dietary components other than probiotics, a clinical trial of 334 schoolchildren revealed that vitamin D supplementation reduced the incidence of influenza A (Urashima *et al.* 2010). It was also reported that taking green tea catechins and theanine reduced the incidence of clinically defined influenza infection in 197 adults (Matsumoto *et al.* 2011). Given those previous studies, our present trial may have been sufficiently large to evaluate the effects of probiotics on influenza, although further clinical trials, such as a randomized, double-blind, placebo-controlled trial, are required to confirm the effectiveness of KB290 against influenza.

A wide variety of exopolysaccharides (EPS) produced by probiotics are suggested to be immunomodulatory agents. Nagai *et al*. (2011) reported that *Lactobacillus delbrueckii* subspecies *bulgaricus* OLL1073R-1 exerted effects against influenza virus in mice and that EPS produced by this strain was one of the active ingredients. It was also reported that bifidobacterial surface EPS was involved in reducing infection by the gut pathogen *Citrobacter rodentium* (Fanning *et al.* 2012). KB290 synthesizes cell-bound EPS, which consists of glucose and *N*-acetylglucosamine, and the EPS plays an important role in bile salt tolerance (Suzuki *et al.* 2013). If the EPS is heterotypic, it might have physiological function such as immunomodulatory effects, as is the case with previously reported heterotypic EPS *in Lactobacillus rhamnosus* GG (Lebeer *et al.* 2011) and *L. delbrueckii* subspecies *bulgaricus* OLL1073R-1 (Makino *et al.* 2006).

In conclusion, we suggest that continual consumption of KB290, isolated from a traditional Japanese pickle ‘Suguki’, in the influenza season could reduce the risk of infection in children. This is believed to be the first report to show reduced incidence of influenza in schoolchildren after consuming a probiotic food. However, further studies are needed to confirm the effectiveness of the probiotic strain KB290, which may be useful in the development of potential anti-influenza agents derived from common foods. In addition, our results indicate that continual consumption of fermented foods containing probiotics may be useful in prevention of influenza.

## Subjects and methods

### Study design

An open-label, parallel-group trial was conducted in two periods, from October 21 to December 13, 2013, and January 14 to March 7, 2014. The study protocol was approved by the Ethics Committee of Kagome Co., Ltd., and carried out in accordance with the International Ethical Guidelines and Declaration of Helsinki.

### Study products

The test drink in this study was produced by Kagome (Nagoya, Japan) and is commercially available in Japan. It contained freshly mixed KB290 at 6 billion colony-forming units and a nutrient mixture (0·6 g protein, <0·3 g fat, 7·6 g carbohydrate, <0·5 g fibre, 8 mg Na, 146 kJ) in one bottle (80 ml). The test drink was delivered to each school every consumption day under refrigeration and stored in a refrigerator until consumption.

### Subjects

Fifteen elementary schools in Nasushiobara city, Tochigi prefecture, Japan, participated in the study. They were divided into Groups A and B to minimize areal and numeral bias. All schoolchildren (6–12 years of age) in the schools were invited to participate in the study. Guardians of children who wished to participate were asked to provide written informed consent. Schoolchildren were excluded if they: (i) had a history of critical illness or were in therapy; (ii) were allergic to milk, soy or apple; (iii) had a history of gastrointestinal surgery (excluding appendicitis); and (iv) lived with employees of Kagome. Schoolchildren who provided written informed consent and did not meet any of the exclusion criteria were identified as eligible.

### Intervention

For the subjects in Group A, the test drink containing KB290 was provided on 5 days per week for 8 weeks in the first period and no treatment in the second period. Subjects in Group B received the drink in the second period but not the first (Fig.2a). Subjects were not recommended to consume the test drink other than when provided at school, because it was commercially available in Japan. If they consumed any extra test drinks, they were required to state this in the questionnaires. They were unrestrained in their daily life including intake of other health-promoting foods.

### Data collection

Guardians of all eligible subjects were asked to submit the questionnaire that consisted of the following questions, after both the first and second periods: (i) how many times per week the subjects consumed the test drink; (ii) whether and when they received influenza vaccine; (iii) whether they were diagnosed with influenza by physicians; and (iv) whether they were diagnosed with common cold or gastroenteritis by physicians. To protect the privacy of the children in the survey, we did not collect information such as age, sex, height and weight. We also asked all participating schools to provide the total number of children diagnosed with influenza during each period.

### Statistical analysis

The incidences of outcomes were compared in the two groups using the chi-square test. Data were analysed using SPSS for Windows ver. 15.0 (SPSS Japan, Tokyo, Japan) and EZR (Saitama Medical Center, Jichi Medical University), which is a graphic user interface for R (The R Foundation for Statistical Computing) (Kanda 2013). *P *<* *0·05 was considered statistically significant.
